# A comparison of oxidative stress in smokers and non-smokers: an *in vivo *human quantitative study of *n*-3 lipid peroxidation

**DOI:** 10.1186/1471-244X-8-S1-S4

**Published:** 2008-04-17

**Authors:** Basant K Puri, Ian H Treasaden, Massimo Cocchi, Sofia Tsaluchidu, Lucio Tonello, Brian M Ross

**Affiliations:** 1 MRI Unit, MRC Clinical Sciences Centre, Imaging Sciences Department, Imperial College London, Hammersmith Hospital, Du Cane Road, London W12 0HS, UK; 2 Three Bridges Medium Secure Unit, West London Mental Health NHS Trust, Uxbridge Road, Southall, Middlesex UB1 3EU, UK; 3 Università di Bologna, Bologna, Italy; 4 Division of Medical Sciences, Northern Ontario School of Medicine, Lakehead University, Room MS 3002, 955 Oliver Road, Thunder Bay, Ontario, Canada P7B 5E1, and Department of Chemistry, Lakehead University, and Public Health Program, Lakehead University, Thunder Bay, Ont., Canada P7B 5E1

## Abstract

**Background:**

Cigarette smoking is believed to cause oxidative stress by several mechanisms, including direct damage by radical species and the inflammatory response induced by smoking, and would therefore be expected to cause increased lipid peroxidation. The aim was to carry out the first study of the relationship of smoking in humans to the level of *n*-3 lipid peroxidation indexed by the level of ethane in exhaled breath.

**Methods:**

Samples of alveolar air were obtained from 11 smokers and 18 non-smokers. The air samples were analyzed for ethane using mass spectrometry.

**Results:**

The two groups of subjects were matched with respect to age and gender. The mean cumulative smoking status of the smokers was 11.8 (standard error 2.5) pack-years. The mean level of ethane in the alveolar breath of the group of smokers (2.53 (0.55) ppb) was not significantly different from that of the group of non-smokers (2.59 (0.29) ppb; *p *= 0.92). With all 29 subjects included, the Spearman rank correlation coefficient between ethane levels and cumulative smoking status was -0.11 (*p *= 0.58), while an analysis including only the smokers yielded a corresponding correlation coefficient of 0.11 (*p *= 0.75).

**Conclusion:**

Our results show no evidence that cigarette smoking is related to increased *n*-3 lipid peroxidation as measured by expired ethane.

## Background

The smoking of cigarettes by humans is believed to cause oxidative stress by several mechanisms, including direct damage by radical species and the inflammatory response induced by smoking [1,2]: peroxyl radicals and reactive nitrogen species cause direct damage, stimulating lipid peroxidation, oxidizing and nitrating proteins, lipids and DNA bases; aldehydes can deplete GSH (reduced glutathione) and modify protein -SH and -NH_2 _groups; cigarette smoke tar phase hydroquinone/quinine complexes diffuse across cell membranes and give rise to semiquinones, superoxide radicals (O_2_^·-^) and hydrogen peroxide (H_2_O_2_); smoking may cause increased phagocytic activity, which in turn leads to increased oxidative stress. Therefore, cigarette smoking would be expected to be associated with increased lipid peroxidation. It is possible to measure lipid peroxidation in humans using a non-invasive sensitive measure of free radical damage by analyzing directly the early products of oxidation in exhalant volatile hydrocarbons [3]. In particular, the volatile hydrocarbon ethane is produced as a terminal catabolite of polyunsaturated fatty acid oxidation and is excreted in the breath. Hence, the level of expired ethane would be expected to be higher in smokers than in age- and gender-matched non-smokers.

However, in a recent study by our group into lipid peroxidation in schizophrenia, while an increased level of expired ethane was found in patients with schizophrenia compared with normal controls, an incidental, and unexpected, finding was that there appeared to be no difference in expired ethane between smokers and non-smokers in the control group [4]. If replicated in a more systematic study of normal controls, this would be an important finding in studies of lipid peroxidation in those psychiatric disorders, such as schizophrenia, in which smoking appears to be particularly common.

We report the first study of the relationship of smoking in humans to the level of *n*-3 lipid peroxidation indexed by the level of ethane in exhaled breath.

## Methods

### Subjects

29 normal control subjects were studied. There was no history of neurological or psychiatric disorder, nor of pulmonary disorders such as chronic obstructive pulmonary disease, in any of the subjects. 11 of the subjects were long-term smokers, while the remaining 18 subjects were non-smokers.

The study was carried out according to the Declaration of Helsinki. The subjects were given both verbal and written details of the study and gave written informed consent. The study was approved by the local research ethics committee.

### Exhalant analysis

Each subject was asked to exhale through a disposable sterile mouthpiece into a syringe (Markes International Ltd., UK) in one long breath, until they were no longer able to exhale any further. This enabled alveolar (end expired) air to be collected from the lungs. The air sample was then injected into an automated thermal desorption tube packed with carbotrap 300 (Perkin-Elmer, UK) via a sodium sulphate drying cartridge (International Sorbent Technology, UK). The air samples were analyzed using a Perkin-Elmer autosystem XL equipped with a turbo mass spectrometer. The automated thermal desorption tubes were desorbed onto the cold trap at 320°C, with the cold trap temperature being held at 5°C. The trap was then rapidly heated to 350°C and the liberated volatiles injected onto a 30 m × 0.32 mm PLOT GQ column (Perkin-Elmer, UK) with helium gas at 2 ml min^-1^. The oven was set at 45°C for 10 min and ramped at 14°C min^-1 ^to 200°C at which temperature it was held for 120 s. Ethane (C_2_H_6_) was eluted at 2.6 min and identified and quantified by mass spectrometry at an *m*/*z *value of 30 by comparison with a standard curve (0–60 pmol) constructed from a C1–C6 alkane standard mix (Supelco, UK).

For the ethane assay, variability and stability data were obtained using a group of 10 controls tested five days in a row with five tubes per test day. Inter-assay variability (as (standard deviation)/mean × 100%) was 17% and intra-assay variability was 10%. The method used was thermal desorption which is a very good way of collecting and immobilizing gases. The gas levels can reduce on the tube owing to chemical instability and simple desorption and diffusion. For the former ethane is a chemically stable molecule but desorption can occur. This was tested by introducing standards in air onto the tubes and testing at various times thereafter. It was found that after one week tubes retained 97% ethane, while retention was 95% after two weeks, and 90% after one month. Therefore the level diminishes over time, but slowly. Our samples were analyzed within one week of collection.

### Statistical analyses

Statistical analyses were carried out using the SPSS version 12 statistics program (SPSS Inc., Chicago).

## Results

### Subjects

There was no significant difference between the smokers and the group of non-smokers in respect of mean age (smokers first: 36.6 (standard error 2.4) years; 34.9 (2.6) years) or gender (five men and six women; eight men and 10 women). The cumulative smoking status for each of the two groups was as follows: smokers mean (S.E.) 11.8 (2.5) pack-years; non-smokers mean (S.E.) 0 (0) pack-years (*p *< 0.00001).

### Expired ethane

**Figure 1:**
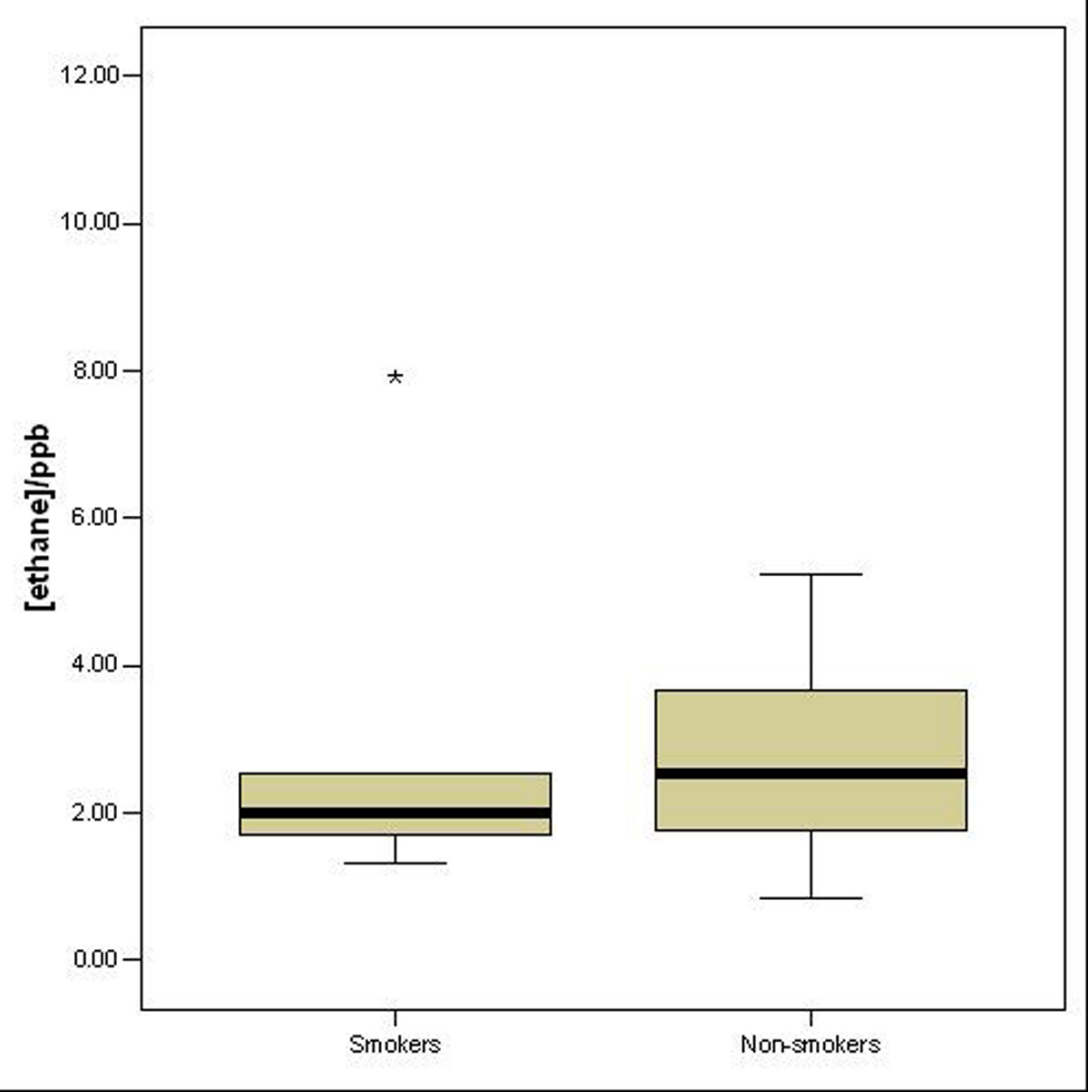
Box-and-whisker plots showing the levels of expired ethane (in ppb) in the two groups. The asterisk in the first group marks a statistical outlier. There was no significant difference in ethane level between the two groups.

As illustrated in Figure 1, the mean level of ethane in the alveolar breath of the group of smokers (2.53 (S.E. 0.55) ppb) was not significantly different from that of the group of non-smokers (2.59 (S.E. 0.29) ppb; *t *= 0.105, *df *= 27, *p *= 0.92).

Further *post-hoc *analyses were carried out to determine whether there was a correlation between ethane levels and cumulative smoking status (measured in pack-years). With all 29 subjects included, the Spearman rank correlation coefficient was -0.11 (*p *= 0.58), while an analysis including only the smokers yielded a Spearman rank correlation coefficient of 0.11 (*p *= 0.75). Thus there was no significant correlation between ethane levels and cumulative smoking status.

## Discussion

This first systematic study of expired ethane in relation to smoking in humans has shown no significant difference between smokers and age- and gender-matched non-smokers, and no significant correlation between expired ethane and cumulative smoking status. These surprising results confirm the earlier indications from our recent study of patients with schizophrenia [4]. There are two sets of findings that may be germane to an understanding of these results.

First, a recent detailed review of oxidative damage to lipids caused by smoking, examining studies which have assayed lipid peroxidation by the thiobarbituric acid (and therefore mainly malondialdehyde) assay, reported highly inconsistent results [5]. It has been generally supposed that such inconsistency might be purely a function of difficulties with this assay, with malondialdehyde being regarded as a relative rather than an absolute marker of lipid peroxidation [1,5].

Second, given that increased lipid peroxidation (and other radical-mediated damage) might be expected to cause some depletion of vitamin E and other antioxidants, it would seem reasonable to expect smoking to be associated with a reduction in such antioxidants. However, it is noteworthy that, while *in vitro *exposure of plasma to cigarette smoke does indeed deplete vitamin E and other antioxidants [2], *in vivo *studies have yielded inconsistent findings, with some studies showing reduced levels of alpha- and gamma-tocopherol in smokers compared with non-smokers [6,7], while at least four others have shown no significant difference between smokers and non-smokers [8-11].

These two sets of findings may be consistent with our results. Although it is difficult to explain, it may be that cigarette smoking is not necessarily associated with increased lipid peroxidation. Further research is clearly required in order, first, to confirm that expired ethane may not be raised in smokers compared with non-smokers, and second, to understand the mechanisms by which cigarette smoking might not lead to increased lipid peroxidation.

## Conclusion

Our results show no evidence that cigarette smoking is related to increased *n*-3 lipid peroxidation as measured by expired ethane.

## Competing interests

The author(s) declare that they have no competing interests.

## Authors' contributions

All the authors made substantial contributions to the design and conception of the study. IHT and BKP were involved in data collection. BMR, IHT and BKP analyzed the data. All authors were involved in the interpretation of the data. All the authors have been involved in drafting and revising the manuscript and have read and approved the final manuscript.

## References

[B1] Halliwell B, Gutteridge JMC (2007). Free Radicals in Biology and Medicine.

[B2] Bruno RS, Traber MG (2006). Vitamin E biokinetics, oxidative stress and cigarette smoking. Pathophysiology.

[B3] Ross BM, McKenzie I, Glen I, Bennett PW (2003). Increased levels of ethane, a non-invasive marker of n-3 fatty acid oxidation, in breath of children with attention deficit hyperactivity disorder. Nutr Neurosci.

[B4] Puri BK, Ross BM, Treasaden IH (2008). Increased levels of ethane, a non-invasive, quantitative, direct marker of *n*-3 lipid peroxidation, in the breath of patients with schizophrenia. Prog Neuropsychopharmacol Biol Psychiatry.

[B5] Lykkesfeldt J (2007). Malondialdehyde as a biomarker of oxidative damage to lipids caused by smoking. Clin Chim Acta.

[B6] Mezzetti A, Lapenna D, Pierdomenico SD, Calafiore AM, Costantini F, Riario-Sforza G, Imbastaro T, Neri M, Cuccurullo F (1995). Vitamins E, C and lipid peroxidation in plasma and arterial tissue of smokers and non-smokers. Atherosclerosis.

[B7] Bolton-Smith C, Casey CE, Gey KF, Smith WC, Tunstall-Pedoe H (1991). Antioxidant vitamin intakes assessed using a food-frequency questionnaire: correlation with biochemical status in smokers and non-smokers. Br J Nutr.

[B8] Lykkesfeldt J, Christen S, Wallock LM, Chang HH, Jacob RA, Ames BN (2000). Ascorbate is depleted by smoking and repleted by moderate supplementation: a study in male smokers and nonsmokers with matched dietary antioxidant intakes. Am J Clin Nutr.

[B9] Dietrich M, Block G, Norkus EP, Hudes M, Traber MG, Cross CE, Packer L (2003). Smoking and exposure to environmental tobacco smoke decrease some plasma antioxidants and increase gamma-tocopherol in vivo after adjustment for dietary antioxidant intakes. Am J Clin Nutr.

[B10] Ross MA, Crosley LK, Brown KM, Duthie SJ, Collins AC, Arthur JR, Duthie GG (1995). Plasma concentrations of carotenoids and antioxidant vitamins in Scottish males: influences of smoking. Eur J Clin Nutr.

[B11] Wei W, Kim Y, Boudreau N (2001). Association of smoking with serum and dietary levels of antioxidants in adults: NHANES III, 1988–1994. Am J Public Health.

